# Zinc Oxide Nanoparticles as Drug Delivery Tools

**DOI:** 10.3390/ma19194121

**Published:** 2026-09-26

**Authors:** Amir Nasrolahi Shirazi, Rajesh Vadlapatla, Ajoy Koomer, Kimberly Tang, Keykavous Parang

**Affiliations:** 1 Department of Pharmaceutical Sciences, College of Pharmacy, Marshall B. Ketchum University, 2575 Yorba Linda Blvd., Fullerton, CA 92831, USA; rvadlapatla@ketchum.edu (R.V.); akoomer@ketchum.edu (A.K.); kimberlytang.cop27@ketchum.edu (K.T.); 2 Center for Targeted Drug Delivery, Department of Biomedical and Pharmaceutical Sciences, Chapman University School of Pharmacy, Harry and Diane Rinker Health Science Campus, 9401 Jeronimo Rd, Irvine, CA 92618, USA; parang@chapman.edu

**Keywords:** Zinc oxide nanoparticles, drug delivery, metal nanoparticles

## Abstract

Zinc oxide nanoparticles (ZnO-NPs) have emerged as promising inorganic nanocarriers for drug delivery due to their unique physicochemical properties, pH-responsive biodegradability, high surface area, and intrinsic biological activity. Their dissolution under acidic conditions enables controlled drug release within the tumor microenvironment and intracellular compartments, improving therapeutic selectivity while aiming to reduce off-target exposure. Surface functionalization with polyethylene glycol (PEG), chitosan, hyaluronic acid, and folic acid has further improved nanoparticle stability, prolonged circulation, and promoted receptor-mediated cellular uptake in targeted tissues. Beyond serving as drug carriers, ZnO-NPs exhibit intrinsic anticancer, antimicrobial, and anti-inflammatory activities, primarily through the generation of reactive oxygen species and controlled release of Zn^2+^ ions, which can contribute to activity in addition to that of the loaded drug. Despite promising preclinical results, challenges continue to limit clinical translation of ZnO-NP-based drug delivery systems, including reproducible large-scale synthesis, long-term colloidal stability and biosafety, pharmacokinetic behavior, and regulatory requirements. Continued optimization of nanoparticle engineering, functionalization, and in vivo validation is essential for advancing ZnO-NPs toward clinical use. This review summarizes the synthesis methods, physicochemical properties, functionalization strategies, and biomedical applications of ZnO-NPs, and discusses remaining challenges and future clinical perspectives.

## 1. Introduction

### 1.1. Inorganic Nanoparticles in Drug Delivery

Inorganic nanoparticles have emerged as versatile platforms for targeted drug delivery. We have previously reviewed this breadth of approaches across multiple inorganic systems. The review on gadolinium-based carriers [1] described hollow gadolinium oxide nanospheres with a spherical morphology and evaluated their potential to deliver bioactive molecules to cells alongside their established role in imaging, positioning gadolinium nanoparticles as dual-purpose theranostic tools rather than purely diagnostic agents. Similarly, the work on iron-based nanoparticles [2] highlighted how core–shell architectures and multifunctional hybrid systems have broadened the therapeutic reach of iron nanoparticles by combining chemotherapy and gene-based approaches within a single platform. Earlier work from our group also explored gold nanoparticle systems, including cyclic peptide-capped gold nanoparticles designed as drug-delivery vehicles [3], reflecting a sustained interest in tailoring the surface chemistry of inorganic nanoparticles to improve cellular uptake and therapeutic loading.

Unlike gadolinium and iron systems, which are valued mainly for imaging or magnetic functionality, and gold and selenium systems, which depend on engineered surface chemistry [4], zinc oxide (ZnO) responds to pH by releasing its payload in acidic microenvironments while also generating reactive oxygen species. This combines passive and active therapeutic mechanisms in a single material [5]. In addition, zinc is an essential element in biological systems, and ZnO has inherent antimicrobial and antioxidant properties. Together, these features have motivated interest in ZnO nanoparticles (ZnO-NPs) as potentially better-tolerated alternatives to other metal-based nanomaterials. Their safety, however, depends on formulation, dose, particle size, route of administration, and duration of exposure.

Beyond gadolinium, iron, and gold, we have also examined selenium as a delivery platform, with a review [6] that describes selenium nanoparticles as versatile delivery tools and detailing surface functionalization strategies, including the use of sulfur-containing amino acids to improve stability and targeting. Taken together, these reviews reflect a consistent research theme: using the physicochemical properties of specific inorganic elements (magnetic behavior in iron and gadolinium systems, surface reactivity in gold and selenium systems) to engineer nanoparticles with tunable release profiles, improved biocompatibility, and multifunctional theranostic capability [7].

While our previous reviews have established how element-specific physicochemical properties enable multifunctional nanocarrier design, ZnO-NPsoccupy a unique position in this landscape. Unlike gadolinium and iron systems, which are valued primarily for imaging or magnetic functionality, or gold and selenium systems, which rely on engineered surface chemistry, the pH-responsive behavior of ZnO triggers-controlled release in acidic microenvironments while simultaneously generating reactive oxygen species, representing a convergence of passive and active therapeutic mechanisms within a single material. Additionally, zinc’s essential role in biological systems, combined with the inherent antimicrobial and antioxidant properties of ZnO, positions these nanoparticles as biocompatible alternatives to precious or toxic metals.

This review synthesizes recent advances in ZnZnO-NP design, examining how morphology, surface modification, and doping strategies can be leveraged to optimize drug loading, cellular targeting, and therapeutic efficacy across multiple disease models. Unlike previously published review papers on ZnO-NPs that focused on synthesis, this review focuses on introducing the various ligands and physical requirements necessary for these nanoparticles to function optimally.

### 1.2. Zinc

Zinc is an essential mineral trace that is involved in various bodily processes. As an enzyme cofactor, zinc is involved in the catalytic activity of more than 300 enzymes in metabolism, digestion, and nerve function [8]. Zinc is also critical for immune function, which is crucial for innate and adaptive immune cells and the body to fight infection, and zinc deficiency is associated with greater susceptibility to illness [9].

In wound healing, zinc is essential for collagen synthesis, cell proliferation, and skin integrity, and it is used in wound treatment and in many wound care products [10]. Zinc is involved in DNA and protein synthesis and is involved in the regulation of gene expression and DNA transcription via zinc finger proteins [11]. Zinc is also an antioxidant that helps stabilize cell membranes and can work with antioxidant enzymes, such as superoxide dismutase, to reduce oxidative stress [12]. Zinc also supports taste and smell, and deficiency is known to cause taste problems such as dysgeusia [13].

### 1.3. Zinc Oxide

Zinc plays several important roles in the body as a trace mineral, but ZnO, the compound most commonly used topically or in nanoparticle systems, behaves in a rather different way. In practice, ZnO remains localized and is not absorbed, distributed and absorbed like dietary zinc (in the skin and in the nanoparticle) as it forms a protective surface barrier with little penetration into deeper tissue, but in nanoparticle or ingested form is stable at neutral physiological pH but dissolves into bioavailable zinc ions (Zn^2+^) in more acidic environments like the stomach (pH = 1.5–3.5), lysosomes (4.5–5.0), tumor microenvironment (pH = 6.5–6.9), and hypoxic tumor environment (5.8–6.5). This pH-dependent dissolution allows ZnO to release zinc where it is needed, feeding into the same basic biological pathways as dietary zinc and also creating reactive oxygen species, which are now being explored for targeted drug delivery [14].

#### 1.3.1. Topical/Skin Barrier

ZnO has been used for decades to protect the skin, particularly in diaper rash creams, calamine lotion, and barrier ointments, and typically with concentrations between 10% and 40%. When applied to the skin, the ZnO particles form a physical, occlusive layer that protects the skin from moisture, irritation, and friction rather than being absorbed systemically. In addition to the mechanical barrier, ZnO has a mild astringent effect and can tighten irritated tissue and reduce localized inflammation, while it has minimal antiseptic action, which prevents microbial colonization of compromised skin. This combination of physical protection and mild anti-inflammatory action explains its long clinical history as a first-line topical agent for diaper dermatitis and other forms of superficial skin irritation [14].

#### 1.3.2. UV Protection

ZnO is one of the two principal inorganic (physical/mineral) UV filters used in sunscreen formulations, the other being titanium dioxide. Unlike organic chemical filters that absorb UV radiation and convert it to heat within the skin, ZnO particles sit on the skin’s surface and primarily protect through the reflection, scattering, and absorption of incident UV photons, with ZnO being particularly effective across the UVA range. This surface-level mechanism reduces the filter’s systemic absorption, which is why mineral sunscreens are commonly recommended for sensitive or pediatric skin. ZnO is also photostable and does not degrade or lose effectiveness after exposure to UV light, unlike chemical filters, which require stabilizing additives to maintain performance over the course of sun exposure [14].

#### 1.3.3. Antimicrobial Activity

ZnO-NPs exhibit broad-spectrum antibacterial and antifungal activity, and their tiny size and relatively high surface area to volume ratio make them even more effective than bulk ZnO. Several mechanisms have been proposed to explain this antimicrobial activity. Particularly, ROS, such as hydrogen peroxide and hydroxyl radicals, are produced at the nanoparticle surface. These ROS damage microbial cell membranes and intracellular components, and Zn^2+^ ions released from dissolving nanoparticles can also disrupt bacterial metabolism and interact with microbial DNA and proteins. Such activity is beneficial for both Gram-positive and Gram-negative bacteria as well as several fungal species, and ZnO-NPs are being investigated as alternatives or as adjuncts to conventional antibiotics in the face of growing antimicrobial resistance [15,16].

## 2. ZnO-NPs-Based Drug Delivery Systems

This review comprehensively examined peer-reviewed scientific literature published between 2013 and 2026. Searches were conducted using PubMed and Google Scholar databases with keywords including “zinc oxide nanoparticles,” “ZnO drug delivery,” “ZnO nanocarriers,” “ZnO functionalization,” “pH-responsive nanoparticles,” and “nanoparticle targeting.” Studies were selected based on inclusion criteria: (1) primary research on ZnO-NPs or nanocomposites; (2) direct relevance to drug delivery, targeting, or therapeutic efficacy; (3) characterization of physicochemical properties (size, morphology, surface modification); and (4) evaluation of biological activity (cell viability, targeting specificity, biodistribution, or in vivo efficacy). Studies that focused exclusively on synthesis without functional evaluation or examined ZnO in contexts unrelated to drug delivery or therapeutics, were excluded.

ZnO-NPs occupy a unique position in inorganic drug delivery: Unlike gadolinium and iron systems (valued primarily for imaging or magnetic properties) or gold and selenium systems (relying on engineered surface chemistry) [17], ZnO combines tunable physicochemical properties with intrinsic pH-responsive behavior and reactive oxygen species generation [18]. This convergence of passive and active therapeutic mechanisms, combined with zinc’s essential physiological role and essential role in human physiology, positions ZnO-NPs as a distinctive platform for precision drug delivery.

The following sections examine how ZnO platform design → drug loading optimization → targeted delivery → enhanced efficacy, with emphasis on how morphology, surface functionalization, and doping strategies control each step of this cascade.

ZnO’s popularity was initially based on its topical, antimicrobial, and UV-protective properties, but upon closer chemical investigation, it was found to be even more attractive for a very different purpose: targeted drug delivery. The same pH-dependent dissolution and ROS generation that made ZnO effective against pathogens on the skin also made it able to drive controlled, site-specific drug release inside the body. This shift in perspective from passive protection to active, stimuli-responsive nanocarrier is what made ZnO-NPs a promising platform for drug delivery research. A schematic illustration of ZnO-NP-based drug delivery systems using various ligands is shown in Figure 1.

ZnO dissolution is strongly pH dependent. At physiological pH (~7.4), dissolution is limited to approximately 10% over several days in cell culture conditions and remains comparably low (<5%) across the mildly acidic extracellular tumor microenvironment (pH 5.8–6.9) in aqueous systems, though dissolution in this range is context-sensitive and can be modulated by proteins, amino acids, and local ionic composition [19,20]. Dissolution accelerates substantially upon cellular internalization: in late endosomal compartments (pH ~5.0–5.5), pronounced acid-triggered Zn^2+^ release has been demonstrated [21], while in lysosomal compartments (pH ~4.5), ZnO undergoes near-complete dissolution within approximately one hour in simulated lysosomal fluid [19,20,22,23]. Importantly, endosomal (pH ~5.0–6.0) and lysosomal (pH ~4.5) compartments represent distinct dissolution environments and should not be treated interchangeably when modeling intracellular Zn^2+^ release kinetics. This property has been utilized to design “smart” stimuli-responsive drug delivery systems in which a chemotherapeutic agent is loaded into or encapsulated within a ZnO carrier and released when the cell reaches an acidic site, thereby preventing early drug leakage [18].

Besides pH dependence, ZnO-NPs offer other advantages for drug delivery as well, such as their dose-dependent toxicity that may be tolerable within a limited window. Simple surface functionalization for targeting ligands and intrinsic cytotoxic effects ROS generation and release of Zn^2+^ that could be combined with a co-delivered drug to act as an anticancer agent [18].

**Biodegradability and Biological Compatibility**

ZnO-NPs degrade into bioavailable Zn^2+^ ions that can be metabolized through normal zinc homeostasis pathways, reducing concerns about non-degradable nanoparticle accumulation inherent to some metal oxide systems [24]. However, this attribute poses a critical stability challenge: premature dissolution in off-target acidic compartments can lead to uncontrolled drug release and localized accumulation of zinc ions in non-target tissues, potentially exceeding safe exposure thresholds [23,25].

**Surface Functionalization Versatility**

The reactive hydroxyl groups on the ZnO surface and exposed zinc ions enable straightforward chemical conjugation of diverse ligands, including polymers, peptides, proteins, and small molecules, without requiring sophisticated linker chemistry often needed for precious metals [26,27,28]. This accessibility facilitates rapid formulation optimization and allows systematic exploration of how surface modifications influence drug loading capacity, targeting specificity, and release kinetics.

**Therapeutic Complementarity**

When combined with chemotherapeutic drugs, ZnO-NPs can contribute therapeutic benefit through mechanisms distinct from the loaded drug, including antimicrobial activity, anti-inflammatory effects, and ROS-mediated cytotoxicity [29,30,31].

This potential for biological complementarity must be carefully weighed against dose-dependent toxicity arising from Zn^2+^ ion release and oxidative stress generation in non-target tissues. This is discussed comprehensively in Section 4.2.

Beyond serving as drug carriers, ZnO-NPs exhibit intrinsic anticancer, antimicrobial, and anti-inflammatory activities, primarily through the generation of reactive oxygen species (ROS) and controlled release of Zn^2+^ ions. These mechanisms operate independently of the loaded drug, potentially complementing drug effects through biological pathways distinct from the primary payload. This represents a distinctive advantage compared to inert nanocarrier platforms (e.g., pure polymeric or lipid carriers) where therapeutic benefit derives entirely from loaded drugs. However, this potential advantage must be carefully distinguished from true synergistic effects, which should only be claimed when experimentally demonstrated through controlled studies comparing (1) free drug alone, (2) ZnO-NPs without drug, (3) drug-loaded ZnO-NPs, with statistical analysis demonstrating greater-than-additive activity [32].

### 2.1. Ligands

Ligands that are conjugated to ZnO-NPs are crucial to their function, stability, and delivery efficiency. These ligands can be easily classified into categories based on their chemical nature, including polymers, peptides and proteins, nucleic acids, small molecules, natural products, polysaccharides, and lipids. In the following section, ligands that are conjuncted with ZnO-NPs are listed and a brief about the procedure was included.

#### 2.1.1. Folic Acid

Folic acid is one of the most commonly used targeting ligands in ZnO-NP-based drug delivery, owing to its high binding affinity for folate receptors, which are drastically overexpressed on the surface of many cancer cell types, such as oral, breast and ovarian cancers, compared to normal tissue.

Folic acid can be conjugated to the ZnO-NP surface via covalent coupling or simple surface adsorption, depending on the desired stability and release profile of the final construct. Upon systemic administration, folate-functionalized ZnO-NPs preferentially accumulate within folate receptor-positive tumor cells via receptor-mediated endocytosis, thereby enhancing both cellular uptake and the cytotoxic efficacy of the encapsulated chemotherapeutic agent while simultaneously reducing nonspecific accumulation in folate receptor-negative healthy tissue [33].

In addition, Gomaa et al. reported the fabrication of ZnO-NP nanocomposites functionalized with folic acid and loaded with Doxorubicin (DOX) (such as ZnONPs/DOX, ZnONPs/FA, and ZnONPs/DOX/FA) that served as effective cancer chemotherapeutic drug delivery systems. They demonstrated that these nanocomposites enhanced anti-tumor and anti-inflammatory activity against Ehrlich ascites carcinoma (EAC) tumor cell lines, both in vitro and in vivo, by suppressing tumor cell proliferation, increasing apoptosis, lowering pro-inflammatory cytokine levels (IL-6 and TNF-alpha), and restoring liver and kidney functions. Additionally, the dual-action system improved the overall safety profile of conventional treatments, such as DOX, by increasing cellular penetration specifically within tumor cells while minimizing off-target toxicity [34].

#### 2.1.2. Synthetic Polymers

Among the various ligands used for ZnO-NP conjugation, polymers are the most widely used because they enhance colloidal stability, inhibit nanoparticle aggregation, and facilitate controlled, prolonged drug release.

##### Polyethylene Glycol (PEG)

PEG is a widely used surface modifier for ZnO-NPs, primarily serving a stabilizing rather than targeting function. In one study, green-synthesized ZnO-NPs (using aloe vera leaf extract) were loaded with DOX, then coated with PEG (MW 400) dissolved in chloroform and combined with the nanoparticles at a 1% *w*/*v* ratio; the mixture was heated at 40 °C under nitrogen for 20 min, then rotary-evaporated and sonicated to drive uniform PEG deposition. PEG attached non-covalently, held in place by electrostatic interactions, hydrogen bonding, and van der Waals forces rather than a chemical linker, forming a “corona” that improved drug-loading capacity, dispersibility, and stealth properties (reduced protein adsorption and immune clearance). The resulting PEGylated DOX-ZnO-NPs showed strong anticancer activity against MDA-MB-231 triple-negative breast cancer cells (IC_50_ = 6.35 μg/mL). Drug-free PEG-ZnO reduced viability to ~50%, while DOX-loaded PEG-ZnO reduced it further to ~35%, reflecting an apparent synergistic effect where ZnO’s intrinsic cytotoxicity (Zn^2+^ release, ROS generation) and loaded DOX reduction act complementarily [35].

##### Polyethyleneimine (PEI)

Polyethyleneimine (PEI) is a cationic, amine-rich polymer used with ZnO-NPs as a capping and stabilizing agent, attaching through electrostatic and coordinative interactions rather than covalent conjugation. In one synthesis, PEI was dissolved directly into a sol–gel reaction medium, where its amine groups coordinated with Zn^2+^ ions during nanoparticle formation, limiting crystal growth and yielding spherical ZnO-PEI nanoparticles roughly 25 nm in size [36].

In another approach, ZnO-NPs were first synthesized separately, then coated post-synthesis with PEI in aqueous medium; the resulting PEI-capped ZnO-NPs were substantially more water-soluble, forming stable colloidal suspensions, and showed enhanced antibacterial activity against multidrug-resistant Gram-negative bacteria compared to uncapped ZnO. PEI’s cationic surface also enables electrostatic loading of negatively charged drugs or nucleic acids, making it useful for combined chemo-gene delivery, though this same cationic character is associated with greater cytotoxicity than neutral coatings such as PEG at higher concentrations [36].

##### Polyvinylpyrrolidone (PVP)

Polyvinylpyrrolidone (PVP) is a hydrophilic, FDA-recognized biocompatible polymer used to coat ZnO-NPs, primarily through non-covalent surface adsorption rather than chemical conjugation. Its polar amide (C=O) groups interact with the ZnO surface via hydrogen bonding and dipole interactions, allowing the polymer to physically adsorb onto and wrap around the nanoparticle [37].

A specific example comes from a chitosan/ZnO–polyvinylpyrrolidone (Cs/ZnO-PVP) nanocomposite, in which PVP was incorporated into the ZnO-containing composite to improve both thermal stability and antibacterial performance compared to uncoated ZnO, demonstrating PVP’s function as a protective, stabilizing coating layer on the ZnO surface. While a dedicated chemotherapeutic drug-loading study specifically using PVP-coated ZnO-NPs was not identified in the literature reviewed here, the expected drug-carrying behavior of such a system can be inferred from a closely related PVP-coated gold nanoparticle system, in which PVP-stabilized gold nanoparticles loaded with DOX (DOX@PVP-AuNPs) achieved significantly greater inhibition of lung cancer cell proliferation than free DOX alone, with enhanced ROS generation and increased tumor suppressor gene expression an effect attributed largely to PVP’s role in improving nanoparticle stability, dispersibility, and cellular uptake rather than to any targeting function of PVP itself [38].

##### Polyvinyl Alcohol (PVA)

PVA is a hydrophilic polymer that coats ZnO-NPs through hydrogen bonding between its hydroxyl groups and the ZnO surface, improving water dispersibility and processability. A specific example is PVA/ZnO nanofiber composite membranes fabricated for surgical gown applications, where PVA’s hydrophilicity improved fiber processability while ZnO provided antibacterial function. Separately, a chitosan–PVA–ZnO nanocomposite conjugated with folic acid (CH/PVA/ZnO-FA) was developed for targeted cancer drug delivery, exhibiting enhanced drug-loading morphology upon the PVA/ZnO combination [39,40].

##### Poly(lactic-co-glycolic acid) (PLGA)

PLGA physically encapsulates ZnO-NPs within its polymer matrix via a solvent/nonsolvent or an emulsion process rather than covalent conjugation, controlling ZnO release while preserving its intrinsic bioactivity. PLGA/nano-ZnO composite particles prepared by a solvent/nonsolvent method demonstrated antimicrobial activity while modulating ZnO release kinetics; a related bimodal PLGA nanocarrier combining ZnO and iron oxide nanoparticles was developed for combined fluorescence/MRI theranostic imaging [41,42].

Ali et al. [43] reported the synthesis and evaluation of an electrospun drug-delivery system composed of a 50:50 polylactic acid (PLA) and gelatin (GA) polymer blend reinforced with 2 wt% ZnO-NPs. The active pharmaceutical ligands, specifically the nonsteroidal anti-inflammatory drugs naproxen (NAP) and meloxicam (MEL), were physically incorporated into the matrix at concentrations of 0.1 wt%, 0.2 wt%, and 0.3 wt%.

Fourier-transform infrared (FTIR) spectroscopy confirmed that ligand encapsulation occurred via physical interactions, retaining characteristic carbonyl (C=O) stretching at 1776 cm^−1^ for NAP and sulfonyl (S=O) stretching at 1161 cm^−1^ for MEL without forming covalent bonds. X-ray diffraction (XRD) analysis indicated that ligand inclusion disrupted polymer crystallinity, resulting in broadened full-width at half-maximum values of 9.38° for NAP and 9.24° for MEL, alongside reduced crystallite sizes (~9 AA) that promoted an amorphous state conducive to controlled drug dissolution. The resulting nanofibrous scaffolds demonstrated high encapsulation efficiency and sustained Fickian diffusion release (*n* < 0.45) over 21 days, achieving maximum cumulative drug release rates of 97.62 ± 4.88% for NAP and 93.75 ± 4.53% for MEL. Additionally, the formulations exhibited potent antibacterial activity against *Staphylococcus aureus* and *Escherichia coli* with inhibition zones up to 18 mm, while maintaining strong biocompatibility toward L929 mouse fibroblasts (81% to 98.5% cell viability) [43].

##### Dextran

Dextran is grafted onto ZnO-NPs (e.g., as dextran-graft-polyacrylamide, D-PAA) to improve aqueous stability and confer selective cancer-cell uptake via endocytosis. D-PAA/ZnO nanosystems were internalized preferentially by prostate and breast cancer cells over non-malignant cells, showing significantly greater cytotoxicity in vitro and in vivo [44].

#### 2.1.3. Natural Polymers

##### Chitosan

Chitosan, a cationic polysaccharide, attaches to ZnO primarily through electrostatic interaction (its protonated amine groups bind the negatively charged/hydroxylated ZnO surface) and can be chemically modified (e.g., *N*-succinylation) for covalent drug conjugation. *N*-Succinyl chitosan-functionalized ZnO-NPs were developed as a pH-sensitive carrier for curcumin, using carbonyldiimidazole-activated carboxyl groups for conjugation. More recently, an exosome-camouflaged chitosan/ZnO/carbon-quantum-dot nanocarrier achieved 88.75% DOX encapsulation efficiency with pH-responsive release and an IC_50_ of 0.8 μM against MCF-7 breast cancer cells [45,46].

Pourmasoumi et al. reported the development of an exosome-camouflaged ZnO-based nanocarrier for pH-responsive DOX delivery. In this system, bone marrow-derived mesenchymal stem cell (BM-MSC) exosomal membranes were employed as a biomimetic surface coating rather than a targeting ligand. The exosomal membrane served primarily as a diffusion barrier and surface-interaction modulator, reducing nonspecific toxicity while enhancing cellular internalization without relying on receptor-mediated targeting [45].

The nanocarrier demonstrated a high DOX encapsulation efficiency of 88.75 ± 2.1% and a drug-loading capacity of 6.8 ± 0.5 wt%. Physicochemical characterization showed that the core nanocomposite (CS/ZnO/CQDs@DOX) had an average hydrodynamic diameter of approximately 180 nm with a positive zeta potential of +30 mV, which increased to approximately 205 nm and decreased to +10.3 mV after exosome membrane coating, confirming successful surface modification. In vitro release studies demonstrated dual-responsive behavior, with accelerated release of DOX and Zn^2+^ under acidic tumor-mimicking conditions (pH 5.4). Approximately 88–90% of the encapsulated DOX was released under acidic conditions, whereas drug release remained significantly lower at physiological pH, indicating effective pH-triggered release. These findings suggest that exosome-coated ZnO nanocarriers offer a promising biomimetic platform for improving tumor-selective drug delivery while minimizing off-target toxicity [45,46].

In another study by Henaish et al., ligand and nanocomposite preparation involved a step-by-step assembly process that combined mebendazole (MBZ), chitosan (Cs) nanoparticles, and ZnO-NPs via precipitation and self-assembly methods. First, low-molecular-weight chitosan nanoparticles (Cs-NPs) were synthesized by dissolving 1 g of chitosan in a 1% *v*/*v* acetic acid solution, adjusting the pH to 5.5–6 using NaOH, and crosslinking dropwise with sodium tripolyphosphate (TPP, 2.5 mg/mL) at a 1:3 *v*/*v* ratio before freeze-drying. The intermediate Cs@MBZ complex was prepared by ultrasonication (30 min) and overnight stirring of 0.03 g Cs-NPs with 0.1 g MBZ drug in 100 mL distilled water, followed by drying at 40 °C [46,47].

Separately, spherical ZnO-NPs (30 nm grain size) were synthesized via a precipitation method by adding 1 M KOH dropwise to 0.2 M Zn(CH_3_COOH)_2_·2H_2_O until a white precipitate formed, which was washed and calcined at 450 °C. Finally, 0.01 g of the prepared ZnO NPs were added to the Cs@MBZ mixture and stirred overnight at room temperature to obtain the full Cs-MBZ@ZnO nanocomposite product, which exhibited physical surface interactions and a grain size of 214 nm for Cs-NPs [47].

##### Gelatin

Gelatin, a hydrophilic protein derived from collagen, interacts with ZnO-NPs through non-covalent physical interactions, primarily hydrogen bonding between gelatin’s amine/hydroxyl side chains and the ZnO surface. In PLA/gelatin/ZnO electrospun nanofibers loaded with naproxen and meloxicam, FTIR confirmed that ZnO interacted with gelatin’s polar groups (–OH, –NH_2_) via hydrogen bonding and van der Waals forces rather than covalent bonding, enhancing hydrophilicity, water absorption, and sustained drug release over 21 days [43].

#### 2.1.4. Peptides

##### RGD

RGD (Arg-Gly-Asp) peptides are covalently conjugated to ZnO nanostructures to target αvβ3-integrin, which is overexpressed on tumor vasculature and certain cancer cells. Green fluorescent ZnO nanowires (ZnO-NWs) were conjugated with the cyclic c(RGDyK) peptide to target αvβ3-integrin, enabling cancer-targeted optical imaging of U87MG human glioblastoma cells with high integrin expression [48].

##### HER2

Short HER2-binding peptides (and HER2-targeted small molecules/drugs) have been conjugated to ZnO-NPs to target HER2-overexpressing breast cancer cells. A 2026 study developed fulvestrant-loaded ZnO-NPs specifically targeting HER2-positive breast cancer and evaluated them using network pharmacology, pharmacogenomics, and in vitro testing [49].

#### 2.1.5. Proteins

##### Albumin

Al-Shehaby et al. reported a study investigating zinc oxide nanoparticles (ZnO-NPs) coated with bovine serum albumin (BSA) and tetraethoxysilane (TEOS) as a targeted drug delivery system for colorectal cancer. The researchers synthesized three nanoparticle formulations: uncoated ZnO, BSA-coated ZnO (ZnOB), and TEOS-coated ZnO (ZnOT) and characterized them extensively using UV-Vis spectroscopy, dynamic light scattering (DLS), FTIR, XRD, and TEM. DLS measurements showed hydrodynamic sizes of 140 ± 13.6 nm for ZnO, 342 ± 8.4 nm for ZnOB, and 145 ± 23.8 nm for ZnOT, while TEM revealed much smaller core diameters (14 ± 5.2 nm, 19 ± 6.8 nm, and 17.8 ± 7.4 nm, respectively), with BSA and TEOS shells measuring 3.6 nm and 5.6 nm. Zeta potential measurements confirmed successful surface modification: ZnO carried a negative charge of 19.21 ± 3.38 mV, ZnOB was closer to neutral at 3.24 ± 4.3 mV, and ZnOT was positively charged at +19.3 ± 4.16 mV, with these charge differences later linked to differences in cellular interaction and toxicity. XRD confirmed the hexagonal wurtzite crystal structure of the ZnO core with high purity, and FTIR verified the presence of amide, NH_2_, and phosphoramide functional groups consistent with BSA attachment.

Al-Shehaby et al. reported that, biologically, the study evaluated cytotoxicity, apoptosis, cell cycle effects, cellular uptake, and mitochondrial function across three cell lines: cancerous Caco-2 (colorectal) cells, HepG2 (liver) cells, and HEK293 (embryonic kidney) cells. All three nanoparticle forms showed selective toxicity toward Caco-2 cells, with IC50 values roughly an order of magnitude lower than in HepG2 cells (ZnO: 219 µg/mL vs. 2080 µg/mL; ZnOT: 235 µg/mL vs. 2178 µg/mL; ZnOB: 308 µg/mL vs. 1912 µg/mL), while HepG2 viability remained near 100% across all tested concentrations, underscoring the nanoparticles’ cancer selectivity. Flow cytometry showed that ZnOB achieved the highest cellular uptake across all cell lines and time points, with fluorescence intensity increasing by more than 80% compared to unmodified ZnO after 24 h, while confocal imaging revealed that unmodified ZnO could penetrate the nucleus (likely via 20–50 nm nuclear pores), whereas the larger ZnOB and ZnOT particles were confined to the cytoplasm. ZnOT induced the strongest apoptotic response, increasing late apoptotic cells by 65% in Caco-2 lines and causing a 40% rise in G2/M phase arrest, while mitochondrial assays showed that unmodified ZnO reduced mitochondrial membrane potential by over 30%, indicating oxidative stress-driven cytotoxicity. Overall, the authors concluded that BSA and TEOS coatings improve nanoparticle stability, reduce nonspecific Zn^2+^ leakage, and preserve selective anticancer activity, positioning ZnOB and ZnOT as promising, more biocompatible alternatives to unmodified ZnO for targeted colorectal cancer therapy, though they noted that in vivo clearance, long-term stability, and immune response remain unresolved before clinical translation [50].

Moreover, Vijayakumar et al. reported that to synthesize egg albumin-doped ZnO-NPs (Ea-ZnO NPs) the chemical precursors for the biogenic ligand and biogenic ligand were prepared through a green synthesis method. The primary precursor was a 0.1 M Zn(CH_3_COO)_2_ solution of Milli-Q water as the metal ion source. For the biological ligand, 5 mL of fresh egg white (albumen) solution (capped and reduced) was directly mixed in 95 mL of prepared zinc acetate solution. The pH of the reaction mixture was then adjusted to 8 by slowly adding a 4% NaOH solution. The reaction mixture was stored in the shaker-heater (200 rpm at 85 °C) at room temperature for 3 h at ambient pressure until all water was destroyed. The thermal response process was controlled to convert intermediate zinc hydroxide (Zn(OH)_2_) nucleation structures into stable and powdery ZnO-NPs bound to egg albumin and produced spherical and hexagonal platelets of 20–60 nm diameter [51].

#### 2.1.6. Monoclonal Antibodies

Hong et al. developed red fluorescent ZnO-NPs conjugated to TRC105, a chimeric monoclonal antibody against CD105 (endoglin), to create a dual-modality PET/fluorescence imaging platform for tumor vasculature targeting. The ZnO-NPs were synthesized by calcination of zinc acetate, 2-aminoterephthalic acid, and acetylenedicarboxylic acid, yielding particles 50–100 nm in size with an unusual, defect-related red fluorescence emission peaking at 598 nm. Surface conjugation proceeded through a multi-step process: the ZnO surface was first thiolated with 3-mercaptopropionic acid, then sequentially PEGylated, functionalized with the chelator NOTA (for radiolabeling), and finally conjugated to thiolated TRC105 via maleimide-thiol chemistry, yielding the final construct NOTA-ZnO-PEG-TRC105 with a hydrodynamic diameter of 101.2 ± 10.7 nm and a zeta potential of −11.2 ± 2.1 mV [52].

After radiolabeling with ^64^Cu (decay-corrected yield of 78.5 ± 10.9%, radiochemical purity >95%), PET imaging in 4T1 tumor-bearing mice showed that TRC105-conjugated ZnO-NPs achieved tumor uptake of 6.4 ± 0.8%ID/g at 0.5 h post-injection, more than three-fold higher than non-targeted ZnO-PEG-NH_2_ controls (1.8 ± 0.6%ID/g), with a strong tumor-to-muscle contrast ratio of 12.1 ± 2.6 at 3 h post-injection. This enhanced uptake was confirmed to be CD105-specific: pre-blocking with excess unlabeled TRC105 (50 mg/kg) significantly reduced tumor accumulation of the TRC105-conjugated nanoparticles (down to 1.7–2.8%ID/g across time points), and ICP-MS analysis of excised tumors corroborated the PET data, showing nearly two-fold higher zinc concentration in tumors treated with the TRC105-conjugated construct (8.3%ID/g) versus the non-targeted control (4.4%ID/g). Histological analysis further confirmed that the TRC105-ZnO conjugate localized specifically to CD31-positive tumor vasculature, with minimal accumulation in muscle tissue. Overall, this study established antibody-conjugated ZnO-NPs as a viable theranostic platform combining quantitative PET imaging with fluorescence-guided detection for tumor-selective drug delivery applications [52].

#### 2.1.7. Aptamers

Han et al. developed aptamer-functionalized ZnO-NPs as a dual-mechanism platform that combines targeted photocatalytic therapy and chemotherapy against cancer cells, addressing the off-target toxicity limitations of conventional cancer treatments. The ZnO-NPs were first functionalized with 3-aminopropyltriethoxysilane (APTES), a silane coupling agent that introduces reactive amine groups onto the ZnO surface. This surface modification was confirmed by FTIR, UV-Vis, and fluorescence spectroscopy, along with TEM, XRD, and dynamic light scattering characterization of the resulting APTES-ZnO-NPs. A targeting aptamer was then conjugated to the amine-functionalized ZnO surface, and fluorescence spectroscopy of the aptamer solution showed a concentration-dependent quenching upon titration with increasing amounts of APTES-ZnO-NPs (from 0 to 1.2 mg/mL), confirming successful aptamer binding to the nanoparticle surface [53].

#### 2.1.8. Miscellaneous

**Liposomes**. The anticancer drug DOX was subsequently loaded onto the aptamer-ZnO construct, with fluorescence quenching of the DOX solution similarly used to confirm drug loading. In vitro cytotoxicity assays demonstrated that this combined platform, leveraging both ZnO’s intrinsic photocatalytic reactive oxygen species generation under irradiation and the chemotherapeutic action of loaded DOX produced a significantly higher rate of cancer cell death compared to either photocatalytic or chemotherapeutic treatment alone. Critically, aptamer functionalization substantially increased nanoparticle accumulation within cancer cells, thereby directly enhancing therapeutic efficacy and demonstrating that aptamer-functionalized ZnO-NPs can serve as a viable platform for combined targeted photocatalytic and chemotherapeutic cancer treatment [54].

Tripathy et al. developed a hybrid drug-delivery nanocomplex in which acid-responsive ZnO-NPs (ZNPs) were physically incorporated into liposomes, rather than conjugated to their surface, to create a multimodal, pH-triggered anticancer platform. The ZnO-NPs were embedded directly within the liposomal structure during formulation, along with the chemotherapeutic drug daunorubicin, yielding spherical ZNP–liposome nanocomplexes with a narrow, uniform size distribution and high drug-encapsulation efficiency. This incorporation strategy exploited ZnO’s well-known acid-sensitive dissolution behavior as a built-in release trigger: under acidic conditions mimicking the intracellular tumor environment, the ZnO-NPs embedded within the liposome rapidly decomposed, disrupting the liposomal structure and accelerating release of the entrapped daunorubicin, whereas the drug remained stably retained without premature leakage under physiological (neutral) pH conditions [54].

Compared with bare (ZnO-free) liposomes, the ZNP–liposome nanocomplexes released their drug cargo significantly faster under acidic conditions, and flow cytometry, confocal microscopy, and MTT assays confirmed that the nanocomplexes were readily internalized by cancer cells, resulting in markedly enhanced cytotoxicity relative to conventional liposomes. Notably, the incorporated ZnO-NPs were multifunctional beyond their role as a pH-trigger: as reported by the authors, ZnO also contributed a synergistic chemo-photodynamic anticancer effect, meaning the nanocomplex combined acid-triggered chemotherapy release with an additional ZnO-mediated phototoxic mechanism. This work illustrates a distinct ZnO integration strategy compared to surface-ligand conjugation approaches; rather than being a target-recognition ligand, ZnO here functions as an embedded, stimuli-responsive structural component that actively controls and enhances the liposome’s own drug-release behavior [54].

**Aloe Socotrina**. Fahimmunisha et al. reported that the biological preparation of Aloe socotrina leaf extract served as the natural reducing and capping ligand matrix for the green synthesis of ZnO-NPs (As-ZnO NPs). The plant extract was prepared by collecting fresh leaves from Karaikudi, Tamil Nadu, India, rinsing them thoroughly with double-distilled water, soaking 10 g of finely chopped leaves in 100 mL of Milli-Q water, boiling the mixture at 70 °C for 20 min, and filtering it through Whatman No. 1 filter paper [55].

For the biosynthesis of the NPs, 10 mL of this aqueous A. socotrina leaf extract was combined with 1 mM of aqueous zinc acetate, and the reaction mixture’s pH was adjusted to 12, yielding a pale white precipitate after stirring. Following centrifugation and washing with distilled water, the resulting pellet was dried in a hot air oven at 80 °C for 3 h and calcined at 350 °C for 3 h to produce the purified As-ZnO NPs. Energy-dispersive X-ray analysis (EDAX) confirmed the success of this capping method, revealing a composition of 78.75% zinc, 21.25% oxygen, and 2.25% carbon as a trace, with the carbon content directly indicating the binding of organic plant phytochemical groups on the nanoparticle surface [55].

**Graphene Oxide**. Additionally, Madeo et al. (2024) reported the development of a ZnO-graphene oxide (Z-G) nanocomposite synthesized via an environmentally friendly, low-temperature hydrothermal process at 100 °C for 6 h followed by calcination at 100 °C for 2 h, yielding a material with a ZnO to graphene oxide (GO) mass ratio of 40:60 as determined by Thermogravimetric Analysis (TGA) [56]. Structural characterization via Powder X-Ray Diffraction (pXRD) revealed an equatorial crystallite size of 11.7 ± 0.1 nm and an axial size of 12.1 ± 0.2 nm, while High-Resolution Transmission Electron Microscopy (HRTEM) confirmed that quasi-spherical ZnO-NPs (5–20 nm) homogeneously coated the thin, wrinkled GO sheets, displaying a mean hydrodynamic diameter of 396 nm in cell culture media via Dynamic Light Scattering (DLS).

The chemotherapeutic agent Paclitaxel (Ptx) was loaded onto this platform at a 0.44 wt% ratio, exhibiting a pH-responsive, sustained release profile under acidic tumor microenvironment conditions (pH 5.5) governed by reversible first-order kinetics (k_R_ = 0.98) with a maximum release (F_max_) of 50% over 48 h. When evaluated against MDA-MB-231 triple-negative human breast cancer cells at a concentration of 25 μg/mL composite (0.110 μg/mL Ptx), the Z-G/Ptx system demonstrated enhanced cytotoxicity by reducing cell viability to 20.4% compared to 45.8% for free Ptx alone (*p* < 0.01), while maintaining high biocompatibility (>97% cell viability) in both unloaded Z-G controls and healthy MCF-10A mammary epithelial cells [56].

**Phenylboronic acid (PBA)**. Mahalanobish et al. (2022) reported that a targeted, pH-responsive nanoformulation was fabricated using 3-carboxybenzeneboronic acid (PBA) as a targeting ligand to deliver the natural flavonoid chrysin [57]. To enable ligand functionalization, amine-conjugated ZnO-NPs (NH_2_-ZnO) were first synthesized using 1 mL of 3-aminopropyltriethoxysilane (APTES). PBA was subsequently tagged to the surface amine groups by activation with 40 mg of 1-(3-dimethylaminopropyl)-3-ethylcarbodiimide hydrochloride (EDC·HCl) and an equivalent amount of *N*-hydroxysuccinimide (NHS) in DMSO for 24 h.

Using a 1:1 feed ratio of ZnO to chrysin, the resulting nanoconjugate (ZnO-PBA-Chry) achieved a drug loading content (DLC) of 30.56% and a drug entrapment efficiency (DEE) of 44.00%. Dynamic light scattering (DLS) measurements confirmed the step-by-step assembly through progressive increases in hydrodynamic diameter, from 142.5 nm for bare ZnO to 285.1 nm for ZnO-PBA and to 415.1 nm for ZnO-PBA-Chry. Surface charge dynamics further demonstrated successful grafting, as the zeta potential shifted from +26.86 mV (amine-functionalized ZnO) to +1.53 mV upon PBA tagging, and finally to −17.36 mV following chrysin complexation. In vitro release testing with a 1 mg/mL chrysin-equivalent nanoparticle suspension demonstrated strong pH-responsive delivery: at acidic tumor microenvironment conditions (pH 5.0), chrysin release reached 28% at 6 h and increased to almost 59% at 48 h, compared to minimal release rates of 14% at pH 6.0 and 9% at physiological pH 7.4 over the same 48 h period [57].

**Eugenol**. Rowhani et al. reported the development of a eugenol-functionalized ZnO-NP system (ZnO@Eug) as a pH-responsive drug delivery platform. Eugenol, a naturally occurring phenolic compound primarily isolated from *Syzygium aromaticum* (clove), was adsorbed onto ZnO-NPs by mixing eugenol dissolved in 70% ethanol with ZnO-NPs under continuous stirring for 24 h in the dark [58].

The resulting nanocomposite exhibited a high ligand loading efficiency of approximately 91–95%, attributed to hydrogen-bonding interactions between the hydroxyl groups of eugenol and the surface hydroxyl groups of ZnO. Successful surface functionalization was confirmed by FTIR spectroscopy via characteristic band shifts, while zeta potential measurements showed a shift from −45 mV for bare ZnO-NPs to −39 mV after eugenol loading, indicating successful surface modification. In vitro release studies further demonstrated pH-responsive, sustained ligand release, with approximately 85% cumulative release at pH 4.5 (mimicking the acidic tumor microenvironment) compared with approximately 55% at physiological pH (7.2) after 72 h. These findings highlight the potential of eugenol-functionalized ZnO-NPs as a stimuli-responsive drug delivery platform capable of preferential payload release under acidic pathological conditions [58].

## 3. Key Physicochemical Properties of ZnO-Based Drug Delivery Systems

The physicochemical characteristics of ZnO-NPs play a critical role in determining their drug-loading capacity, colloidal stability, biological interactions, and overall therapeutic performance. Parameters such as particle size, morphology, surface charge, crystallinity, porosity, and surface functionalization significantly influence drug encapsulation efficiency, release kinetics, biodistribution, pharmacokinetics, cellular uptake, and intracellular trafficking. Careful optimization of these properties is therefore essential for improving the safety, targeting capability, and therapeutic efficacy of ZnO-NP-based drug delivery systems, making them promising nanocarriers for precision medicine applications [59]. Figure 2 demonstrates factors governing the therapeutic performance of ZnO-NPs-based drug delivery systems.

### 3.1. Particle Size and Morphology

The physicochemical properties of ZnO-NPs, particularly particle size and morphology, are critical determinants of their performance as drug delivery vehicles, directly influencing drug loading, colloidal stability, cellular internalization, biodistribution, and therapeutic efficacy. Current evidence indicates that nanoparticles with diameters ranging from 20 to 100 nm are generally the most suitable for systemic drug delivery, facilitating passive tumor accumulation through the enhanced permeability and retention (EPR) effect while minimizing rapid renal clearance and excessive uptake by the mononuclear phagocyte system [59,60].

However, the optimal size range is narrower and more application-dependent than a simple “under 100 nm” rule suggests. The best range for intravenous administration is between 2 and 200 nm, as particles larger than 200 nm are very quickly phagocytically cleared by macrophages and cannot take advantage of EPR. In this range, more granular data are available: particles from 100 to 200 nm have been shown to have up to 4 times the uptake of tumors, compared to particles larger than 300 nm or smaller than 50 nm, which is truly the sweet spot for passive tumor accumulation [59].

At the cellular level, a different optimum is widely accepted across most in vitro studies: maximum cellular uptake in non-phagocytic cells typically occurs in the 10 to 60 nm range and is not related to particle core composition or surface charge. ZnO-NPs from 30 to 70 nm are found to be most often more uptake-dependent and more effective for intracellular trafficking.

This creates a practical design tension for ZnO-specific carriers, since the size that maximizes tumor-level accumulation via EPR (100–200 nm) differs from the size that maximizes individual cell uptake (30–70 nm). A directly comparable example illustrating this tradeoff is seen in BSA/TEOS-coated ZnO-NPs, where a smaller, positively charged variant (145 ± 23.8 nm, zeta potential +19.3 ± 4.16 mV) was more efficiently internalized via endocytosis due to its favorable size for membrane interaction. On the other hand, a larger variant (342 ± 8.4 nm) instead showed enhanced retention at the target site due to slower clearance, demonstrating that “optimal” size in ZnO drug delivery is not a single fixed value but a tradeoff between faster cellular uptake and prolonged local retention, depending on the therapeutic goal. In addition to particle size, narrow size distribution and high colloidal stability are essential for maintaining reproducible biological behavior and preventing aggregation under physiological conditions. Consequently, surface functionalization with stabilizing ligands, including polyethylene glycol, chitosan, polyvinylpyrrolidone, and hyaluronic acid, is commonly employed to preserve nanoparticle dispersion, improve biocompatibility, and optimize pharmacokinetic properties [50].

The morphology of ZnO-NPs plays an equally pivotal role in determining drug delivery performance by influencing surface area, drug-loading capacity, dissolution behavior, and cellular interactions. ZnO-NPs have been produced in a variety of shapes such as spherical particles, nanorods, nanowires, nanoflowers, nanosheets, and hollow or mesoporous nanostructures, which are all characterized by their different physicochemical and biological properties [23].

Spherical ZnO-NPs are the most widely used in drug delivery because they offer uniform drug distribution, favorable colloidal stability, and efficient cellular uptake while minimizing nonspecific interactions with biological tissues [50,59].

In contrast, anisotropic particles such as nanorods and nanoflowers have more specific surface areas for drug loading and can affect cellular biological activity due to their larger aspect ratios. Since rod-shaped ZnO-NPs achieve superior cellular absorption compared to spherical particles of similar volume, the contact surface area of the particles is larger and more likely to be absorbed by them with the lipid bilayer than spherical ZnO particles, and therefore they are concentrated in the spleen and kidneys and not in the liver. Hollow and mesoporous ZnO nanostructures have emerged as promising alternatives because their internal cavities enable higher drug encapsulation efficiencies and pH-responsive release following ZnO dissolution within the acidic tumor microenvironment [59,60,61].

Taken together, optimizing both particle size and morphology and coordinating these choices with the intended route of administration and therapeutic target is essential for maximizing the therapeutic potential of ZnO-NP-based drug delivery systems while maintaining favorable safety and pharmacokinetic profiles.

### 3.2. Surface Functionalization and Encapsulation

The role of surface functionalization was discussed in detail in the previous section, “Ligands,” which described the ligands conjugated to ZnO-NPs and their effects on the physicochemical, biological, and therapeutic properties of the nanoparticles. Surface chemistry represents the single most adaptable strategy for enhancing the performance of ZnO-NPs as drug carriers. Because unmodified ZnO-NPs tend to aggregate and undergo rapid opsonization in biological fluids, surface engineering is routinely applied to achieve four key objectives: stabilizing the nanoparticle colloid, extending circulation time in the bloodstream, enabling selective targeting of specific cells or tissues, and introducing stimuli-responsive drug release behavior [50,62].

### 3.3. Common Loading Strategies and Their Implications

Drug loading is a defining step in the design of ZnO-NP-based delivery systems, as the loading mechanism directly determines drug-carrier stability, release kinetics, and ultimately therapeutic performance. Four principal strategies are used in the literature (Table 1): physical adsorption at the surface, coordination/complexation with surface Zn^2+^ ions, covalent conjugation, and encapsulation within a surrounding matrix. Each is described below, followed by a comparative summary of its respective advantages, limitations, and best-fit applications.

#### 3.3.1. Physical Adsorption

Physical adsorption is the simplest loading route: the drug is mixed with pre-formed ZnO-NPs and held at the surface by van der Waals forces, electrostatic attraction, and hydrogen bonding, without chemically modifying either the drug or the carrier. The hydroxyl-rich ZnO surface favors molecules with complementary polar groups; for example, eugenol adsorbed onto ZnO-NPs achieved ~91–95% loading efficiency through hydrogen bonding, with ~85% release at pH 4.5 versus ~55% at pH 7.2 over 72 h [58]. The latter figure illustrates the main drawback: weakly bound cargo can be displaced by dilution or competing proteins and ions, causing burst release and premature leakage (Table 1). Physical adsorption is therefore best suited to applications where simplicity matters more than release control.

#### 3.3.2. Coordination/Complexation with Zn^2+^

A loading mechanism more specific to ZnO than to most other inorganic nanocarriers involves direct coordination bonding between the drug molecule and surface zinc ions. In one representative system, DOX was loaded onto amino-terminated ZnO-NPs via coordination bonding with surface Zn^2+^, while a co-delivered nitric oxide donor was separately attached through covalent amide bonding, producing dual-drug nanospheres approximately 7.9 nm in size [63].

Because this coordination bond is inherently pH-sensitive, drug release becomes intrinsically coupled to ZnO’s own acid-triggered dissolution behavior. This is a key mechanistic advantage: it allows the nanoparticle to function as a self-triggering release system in acidic environments such as the tumor microenvironment or endolysosomal compartments, without requiring an additional externally engineered stimuli-responsive coating [64].

#### 3.3.3. Covalent Conjugation

Where more precise, stable, and sustained drug attachment is required, drugs (or reactive linker groups) can be covalently bonded to a functionalized ZnO surface for example, via amide bond formation to amine-terminated ZnO, or through silane-based surface chemistry (e.g., aminopropyl groups) that introduce reactive conjugation sites [65].

Covalent loading generally provides greater drug retention and more predictable release kinetics than physical adsorption, since the drug cannot dissociate through simple dilution or competitive displacement in biological fluids. The main limitations of this method are the synthetic complexity and the risk that conjugation chemistry could chemically change the drug’s active site.

This was illustrated in a carfilzomib-loaded ZnO nanocrystal study, in which biological testing showed that the drug retained its original mechanism of action in a number of myeloma cell lines despite covalent surface attachment. Mishra et al. (2022) reported that the anti-cancer drug DOX was conjugated into the biogenically synthesized ZnO-NPs by 1-ethyl-3-(3-dimethylaminopropyl) carbodiimide (EDC) as an activator [66]. In a 1 mL reaction with 50 mM HEPES buffer, 50 µg of DOX, 600 µL of ZnO-NPs, and 5 mM EDC, the free amino group of DOX was directly bound to the carboxylate groups on the surface of the nanoparticles, and the reaction was done at a constant temperature of 30 °C for 5 h. Following the incubation period, the resulting DOX-loaded ZnO-NPs (DOX-ZnONPs) were isolated from the mixture via centrifugation at 10,000 rpm for 10 min for subsequent characterization. Table 1 elaborates on four loading strategies using ZnO nanoparticles.

#### 3.3.4. Encapsulation Within a Polymer or Composite Matrix

Rather than binding the drug directly to the ZnO surface, this strategy physically embeds both the ZnO-NP and the drug within a surrounding carrier matrix, for example, a polymer coating, porous shell, or composite nanofiber scaffold.

Representative examples include chitosan-encapsulated ZnO quantum dots [67,68,69] and PLA/gelatin/ZnO composite nanostructures [43,51]. Encapsulation with a hydrophilic, cationic coating, such as chitosan, has been shown to enhance the colloidal stability of ZnO quantum dot carriers [67,68,69,70].

Functionally, this approach decouples drug release from ZnO’s intrinsic dissolution chemistry, introducing an additional, independently tunable release mechanism, such as diffusion through the matrix, matrix degradation, or matrix swelling, layered on top of ZnO’s native pH-responsiveness. This generally produces more gradual, sustained-release profiles, at the cost of greater formulation complexity due to the additional matrix component [71]. 

## 4. Challenges and Limitations

Despite the considerable promise demonstrated by ZnO-NP-based drug delivery systems across preclinical studies, several interconnected barriers continue to limit their translation from bench to bedside. These challenges span the biological, toxicological, manufacturing, and regulatory domains, and must be addressed collectively rather than in isolation for ZnO-NPs to achieve broader clinical adoption.

### 4.1. Stability in Biological Environments

A central challenge for ZnO-NP-based carriers is maintaining colloidal and chemical stability once introduced into complex biological fluids. Unmodified ZnO-NPs are prone to aggregation in physiological media due to their high surface energy and tendency toward opsonization, which can alter their effective size, reduce circulation time, and diminish targeting precision [50].

Compounding this issue, the very property that makes ZnO attractive for stimuli-responsive drug delivery, its pH-dependent dissolution into Zn^2+^ ions, also represents a stability liability outside of the intended target environment. Premature dissolution in blood, interstitial fluid, or off-target acidic compartments can result in uncontrolled drug release, reduced therapeutic payload at the intended site, and localized accumulation of zinc ions in non-target tissues.

Reproducibly maintaining a stable, non-aggregated colloidal suspension over clinically relevant timeframes remains difficult, particularly for green-synthesized formulations, where batch-to-batch variability in capping biomolecules can produce inconsistent surface coatings and, consequently, inconsistent dissolution kinetics. Achieving a formulation that is stable during storage and circulation, yet responsive on demand at the target site, therefore continues to represent one of the most significant unresolved engineering tradeoffs in ZnO-NP drug delivery design.

### 4.2. Immunogenicity and Toxicity Concerns

While ZnO is classified by the FDA as “Generally Recognized as Safe” (GRAS) in bulk form for topical and dietary applications, this regulatory designation does not automatically extend to ZnO-NPs [72,73]. The toxicological behavior of nanoscale ZnO differs fundamentally from bulk ZnO due to (1) dramatically increased surface area-to-volume ratio enabling greater Zn^2+^ bioavailability; (2) enhanced cellular internalization of nanoparticles (<200 nm) relative to bulk particles; (3) accumulation in acidic intracellular compartments where dissolution and ion release are accelerated; and (4) systemic biodistribution to organs following intravenous administration, versus local topical application of bulk ZnO [74,75,76].

Toxicity is strongly dose-, size-, and morphology-dependent, and is primarily mediated through the release of Zn^2+^ ions, which can induce oxidative stress, mitochondrial dysfunction, DNA damage, and, at sufficiently high concentrations, apoptosis or necrosis in both cancerous and healthy cells [77].

**Dose-Dependence**

In vitro cytotoxicity of ZnO-NPs is concentration-dependent, but the threshold varies with cell type, particle dispersion, and the endpoint measured. In primary human nasal mucosa cells, ZnO-NPs reduced viability at 50 µg/mL, increased DNA damage from 10 µg/mL, and raised IL-8 secretion from 5 µg/mL, whereas ZnO powder showed no cyto- or genotoxicity in the same assays [78]. Comparisons with larger particles are less clear-cut in other systems. In human colon-derived RKO cells, nanosized ZnO was roughly twofold more potent than micrometer-sized ZnO (LC_50_ 15 vs. 29 µg/cm^2^), although both were cytotoxic [79]. In human bronchial BEAS-2B cells, nanosized and fine ZnO were toxic within a similar narrow range after 48 h (1.5–3.0 µg/cm^2^), and differences appeared mainly in short-term DNA damage and depended on dispersion status [80]. In fish cell lines, ZnO-NP cytotoxicity exceeded that of bulk ZnO and Zn^2+^, and oxidative stress increased with dose [81]. Mechanistically, direct particle–cell contact rather than dissolved Zn^2+^ appeared to drive toxicity in RKO and fish cells [79,81], and TEM showed particles in the cytoplasm of only ~10% of nasal cells and in the nucleus of 1.5% [78]. Overall, nanoscale ZnO is generally more potent per unit mass than bulk ZnO, but bulk ZnO is not inert, and small differences in dispersion can shift the apparent toxic threshold [80].

**Size-Dependence**

Smaller ZnO-NPs (<50 nm) demonstrate significantly greater cellular uptake and higher cytotoxic potency than larger particles (>100 nm), independent of surface coating or targeting ligand. This size dependence reflects both increased surface area per unit mass and more efficient cellular internalization via pinocytosis and endocytosis [82,83,84,85].

**Morphology-Dependence**

Rod-shaped and flower-shaped ZnO-NPs exhibit greater cellular toxicity than spherical particles of equivalent core volume, likely due to their larger surface areas and potentially sharper edges enabling more efficient cellular membrane interaction. This suggests that morphology is as critical a safety parameter as size and composition [86,87].

**Organ-Specific Accumulation and Chronic Toxicity**

Following systemic (intravenous or oral) administration, ZnO-NPs preferentially accumulate in highly perfused, phagocyte-rich organs: the liver, spleen, and lungs. Chronic or repeated dosing therefore raises legitimate concerns about cumulative organ-specific toxicity, particularly hepatotoxicity (elevated liver enzymes, altered metabolic function) and pulmonary inflammation (alveolar macrophage activation, inflammatory cytokine release) Long-term in vivo toxicity data specifically examining repeated-dose toxicity, bioaccumulation in organs, and potential chronic effects remain limited and represent a critical gap for clinical translation [24,88,89,90].

Because ZnO-NPs accumulate preferentially in highly perfused, phagocyte-rich organs such as the liver, spleen, and lungs following systemic administration, chronic or repeated dosing raises legitimate concerns about cumulative organ-specific toxicity, particularly hepatotoxicity and pulmonary inflammation [91].

Beyond direct cytotoxicity, immunogenicity remains a comparatively underexplored dimension of ZnO-NP safety: surface-adsorbed proteins forming a “protein corona,” along with certain surface ligands (e.g., antibodies, polymeric coatings), can trigger unwanted immune recognition, complement activation, or hypersensitivity reactions, potentially accelerating nanoparticle clearance or provoking inflammatory responses that compromise both safety and therapeutic efficacy.

The absence of standardized, harmonized toxicological testing protocols across studies, which vary widely in animal models, routes of administration, dosing regimens, and nanoparticle characterization methods, further complicates efforts to establish reliable safety thresholds and No Observed Adverse Effect Levels (NOAELs) applicable across different ZnO-NP formulations.

### 4.3. Scalability and Reproducibility

An obstacle to translate laboratory-scale ZnO-NP synthesis into large batch production in clinical and commercial applications is that, while green synthesis methods (as reported environmental advantages) are based on biological precursors (plant extracts, bacteria or fungi) that can be derived from multiple sources (and/or seasons), which in turn can change from one batch to the next with respect to particle size, shape, surface chemistry and drug loading capabilities [92].

Since the biological performance of ZnO-NPs is highly sensitive to these physicochemical factors, a minor disturbance in synthesis can result in unpredictable drug delivery. The problem of reproducibility resurfaces during the surface functionalization phase, as conjugation processes for ligand attachment, PEGylation, or covalent drug loading are not tightly controlled, leading to variable batch yields and/or conjugation efficiency. To address these problems, we need standardized, automated, and continuous-flow manufacturing platforms and similar characterization methods (e.g., DLS, TEM, FTIR, and zeta potential analysis) to test formulations in early studies that can be reliably reproduced for clinical trials and eventual commercial production.

### 4.4. Regulatory and Translational Barriers

The regulatory landscape for nanomedicine products, such as ZnO-NP-based drug delivery systems, is still fragmented and lacks a globally harmonized approval pathway. Nanoparticles do not fit neatly into conventional pharmaceutical regulatory frameworks because their physicochemical and biological properties, and consequently their safety and efficacy profiles, can change substantially with relatively minor formulation adjustments (e.g., surface coating, size, or morphology), unlike small-molecule drugs, where structure–activity relationships are typically more predictable [92,93].

Regulatory agencies such as the FDA and EMA generally apply a risk-based, case-by-case evaluation approach for nanomedicines, requiring comprehensive documentation of synthesis methods, physicochemical characterization, and both in vitro and in vivo safety and performance data; however, the absence of a unified global framework can create inconsistent requirements across jurisdictions, prolonging development timelines and complicating multinational clinical trials [94].

Compounding these regulatory hurdles is a persistent shortage of comprehensive, long-term in vivo toxicity and pharmacokinetic data for ZnO-NPs specifically, which limits the evidentiary basis regulators can draw on when evaluating new formulations. Early and proactive engagement with regulatory bodies through structured programs (e.g., the FDA’s Emerging Technology Program or the EMA’s Innovation Task Force), alongside continued investment in standardized preclinical testing protocols, will likely be necessary to reduce uncertainty and accelerate the clinical translation of ZnO-NP-based therapeutics.

## 5. Future Perspectives

### 5.1. Toward Precision and Personalized Nanomedicine

The next generation of ZnO-NP-based drug delivery is likely to move beyond generalized, “one-formulation-fits-all” designs toward nanocarriers engineered for specific patient populations, tumor subtypes, or disease states. Given that receptor expression patterns (e.g., folate receptors, HER2, CD44, or integrin subtypes) vary considerably among individual tumors and patients, future ZnO-NP platforms may increasingly incorporate patient- or biomarker-specific targeting ligands selected based on individual tumor profiling, rather than relying on a single universal targeting strategy.

Precision-medicine approaches may also extend to tailoring nanoparticle size, morphology, and surface chemistry to the specific pharmacokinetic requirements of a given indication, for instance, favoring smaller, rapidly internalized ZnO-NPs for aggressive, rapidly proliferating tumors, versus larger, retention-optimized formulations for indications where prolonged local drug exposure is prioritized.

The integration of computational modeling and artificial intelligence-based design tools that can predict interactions between nanoparticles and biological systems prior to synthesis may accelerate this process by enabling scientists to rigorously design ZnO-NP formulations for each patient or tumor, rather than relying on trial and error.

### 5.2. Engineering Smart and Hybrid ZnO Nanocomposites

A clear trend in recent literature is towards increasingly sophisticated, multifunctional ZnO-based nanoplatforms that combine several therapeutic and diagnostic applications in a single structure. This development is ongoing for hybrid systems that combine ZnO with other functional materials, such as iron oxide (for imaging and magnetically guided delivery), gold or other plasmonic nanoparticles (for photothermal therapy), or polymeric and liposomal matrices (for layered, independently tunable release control) [95].

Emerging “nanozyme” approaches, in which ZnO-NPs are engineered to exhibit catalase- or peroxidase-mimicking enzymatic activity, represent another promising direction, enabling ZnO-NPs to actively modulate the tumor microenvironment (e.g., by depleting hydrogen peroxide or generating cytotoxic reactive oxygen species on demand) rather than functioning purely as a passive drug carrier [96].

Similarly, growing interest in combining ZnO’s intrinsic acid-triggered dissolution with externally applied stimuli, such as near-infrared light for combined photodynamic/photothermal therapy, or ultrasound for on-demand triggered release, suggests that future ZnO-NP platforms will increasingly function as multimodal, externally controllable therapeutic systems rather than relying solely on the tumor microenvironment’s intrinsic acidity as the sole release trigger [97].

Additionally, there is growing recognition that ZnO-NPs can influence antitumor immune responses beyond direct cytotoxicity, including the induction of immunogenic cell death and modulation of the immunosuppressive tumor microenvironment, opening a promising avenue for combining ZnO-NP-based drug delivery with immunotherapeutic strategies [98].

### 5.3. Integrating Regulatory, Safety, and Clinical Frameworks

Advancing ZnO-NP-based drug delivery toward clinical use will require closer, earlier collaboration between researchers, industry, and regulatory bodies that have historically characterized nanomedicine development. Priority areas include the development of standardized characterization and toxicological testing protocols for metal oxide nanoparticles, which would enable more direct comparison of safety and efficacy data across independent studies and reduce the current fragmentation in dosing regimens, exposure durations, and animal models used across the literature.

Establishing consensus-based safety thresholds (e.g., harmonized NOAEL values) for ZnO-NPs across different routes of administration and indications would similarly streamline regulatory review. Greater emphasis on long-term in vivo pharmacokinetic and biodistribution studies, ideally supported by advanced analytical techniques such as LC-MS/MS and real-time in vivo tracking, will be necessary to close the current knowledge gaps around chronic exposure and cumulative organ toxicity.

Finally, closer alignment with international harmonization efforts (such as the ICH) could help reduce the duplicative regulatory burden across jurisdictions and accelerate the pathway from preclinical validation to first-in-human clinical trials for ZnO-NP-based therapeutics.

### 5.4. Outlook

Together, ZnO-NPs have a unique position in the field of inorganic drug delivery platforms, as they carry intrinsic, pH-driven anticancer activity. However, a long history of topical and dietary use in bulk form does not automatically extend to nanoscale ZnO. A wide range of surface functionalization properties that have also evolved (e.g., cancer therapy, antimicrobial therapy, and wound healing) in an analogous way with the development of cancer therapy, antimicrobial treatment, and wound healing.

The development of our field over the years, from passive drug carriers to more complex, stimulus-driven, and immune-responsive drug delivery platforms, indicates that ZnO-NPs will play an important role in precision nanomedicine. However, their clinical application will be achieved if the field can overcome the stability, toxicity, manufacturing, and regulatory issues above through coordinated cooperation. With continued investment in standardization, long-term safety data generation, and rational formulation design, as well as sensible and patient-oriented design of drug delivery systems, ZnO-NPs could be the next step from a promising experimental platform to a clinically validated tool for targeted, controlled treatment.

## 6. Conclusions

ZnO-NPs are a versatile and mechanistically distinct platform for targeted drug delivery, offering unique bioactivity, pH-dependent dissolution, and a variety of surface functionalization opportunities that few other inorganic nanocarriers can compete with.

ZnO-NPs can be produced using a wide range of ligand classes from small molecules such as folic acid to synthetic and natural polymers, peptides, proteins, monoclonal antibodies, and aptamers, offering varying stability, specificity, and control of release.

The physicochemical properties of the nanoparticle, particularly drug-loading efficiency, cell uptake, and biodistribution, can also affect its formulation, which is why the design is meant for an application-specific rather than a one-size-fits-all approach. Despite the obvious promise of the ZnO-NP drug, several barriers to commercialization remain: colloidal and chemical stability in biological environments, uncharacterized immunogenicity and dose-dependent toxicity, manufacturing and reproducibility problems, and a regulatory framework that has not yet fully adapted to the unique properties of nanoscale therapeutics. These barriers will need to be addressed through interdisciplinary interaction between materials science, pharmacology, toxicology, and regulatory science. With progress in these areas, ZnO-NPs are well-positioned to progress from a well-studied experimental platform into a clinically validated component of precision, targeted drug delivery.

Together, ZnO-NPs demonstrate promise as an alternative to precious or toxic metals when appropriately formulated and administered at safe dose levels. However, clinical application will require overcoming significant challenges in nanoscale toxicity assessment, biodistribution control, long-term safety validation, and regulatory harmonization. These barriers are surmountable through coordinated investment in standardized preclinical testing, comprehensive in vivo safety studies, and proactive engagement with regulatory agencies.

## Figures and Tables

**Figure 1:**
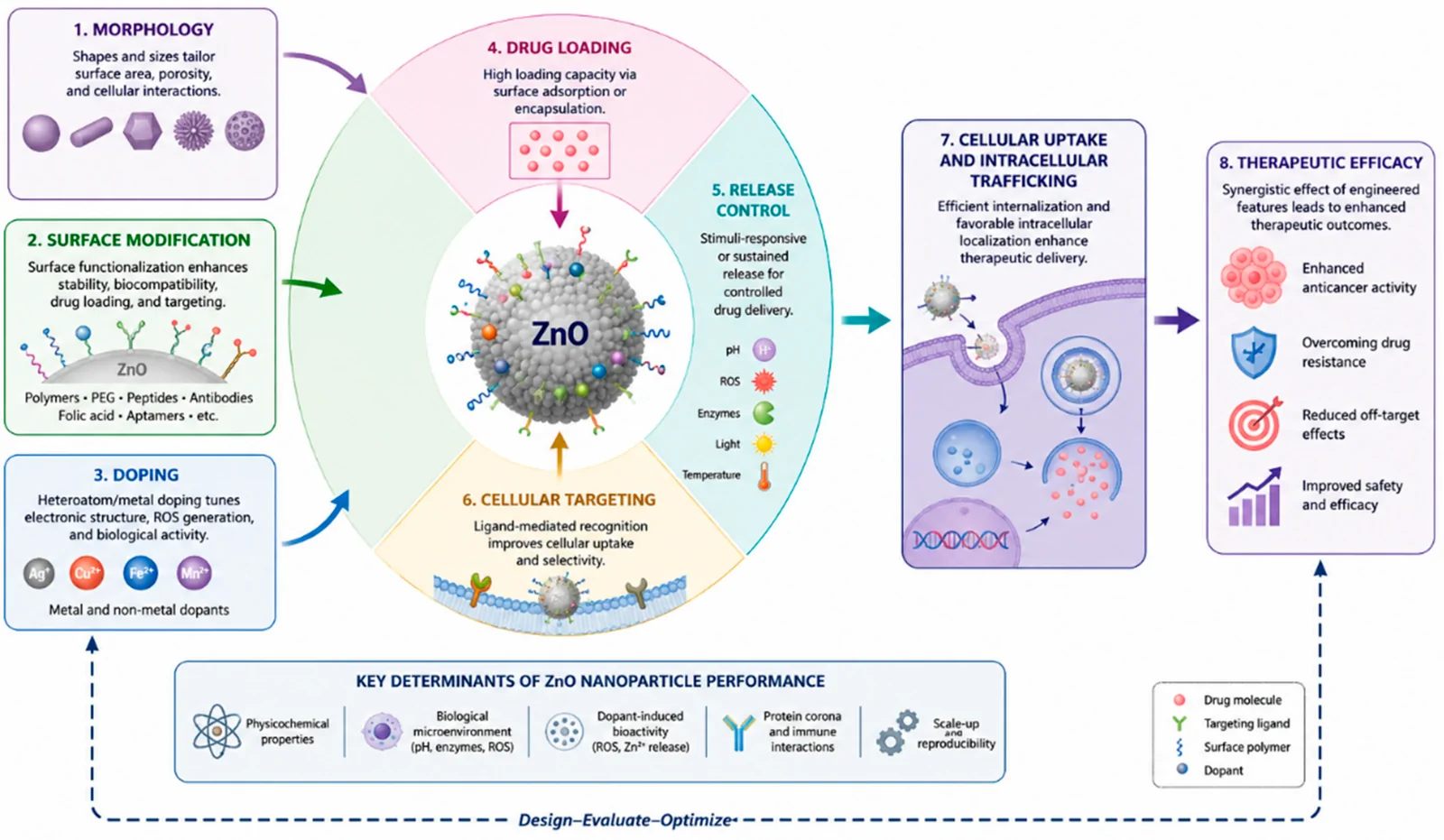
ZnO nanoparticle engineering for Optimizing therapeutic efficacy. Schematic overview of zinc oxide (ZnO) nanoparticle–based drug delivery systems illustrating the interplay among particle morphology, surface engineering, doping, drug loading, cellular trafficking, and biological fate in cancer therapy. The physicochemical and biological properties of ZnO nanoparticles can be modulated through the control of particle morphology, surface functionalization, and heteroatom or metal doping. These engineering strategies can influence surface area, drug-loading capacity, cellular interactions, electronic structure, reactive oxygen species (ROS) generation, Zn^2+^ release, and overall biological activity. Drugs can be incorporated through surface adsorption or encapsulation and subsequently released through sustained or stimuli-responsive mechanisms triggered by pH, ROS, temperature, light, or enzymes. Ligand-mediated surface recognition can promote selective cellular uptake and intracellular trafficking, facilitating delivery to desired cellular compartments. The coordinated integration of these features can enhance drug delivery, therapeutic efficacy, and treatment outcomes. Overall, the figure highlights the multifunctional and stimuli-responsive nature of ZnO nanoparticles as tunable nanocarriers for targeted cancer therapy.

**Figure 2:**
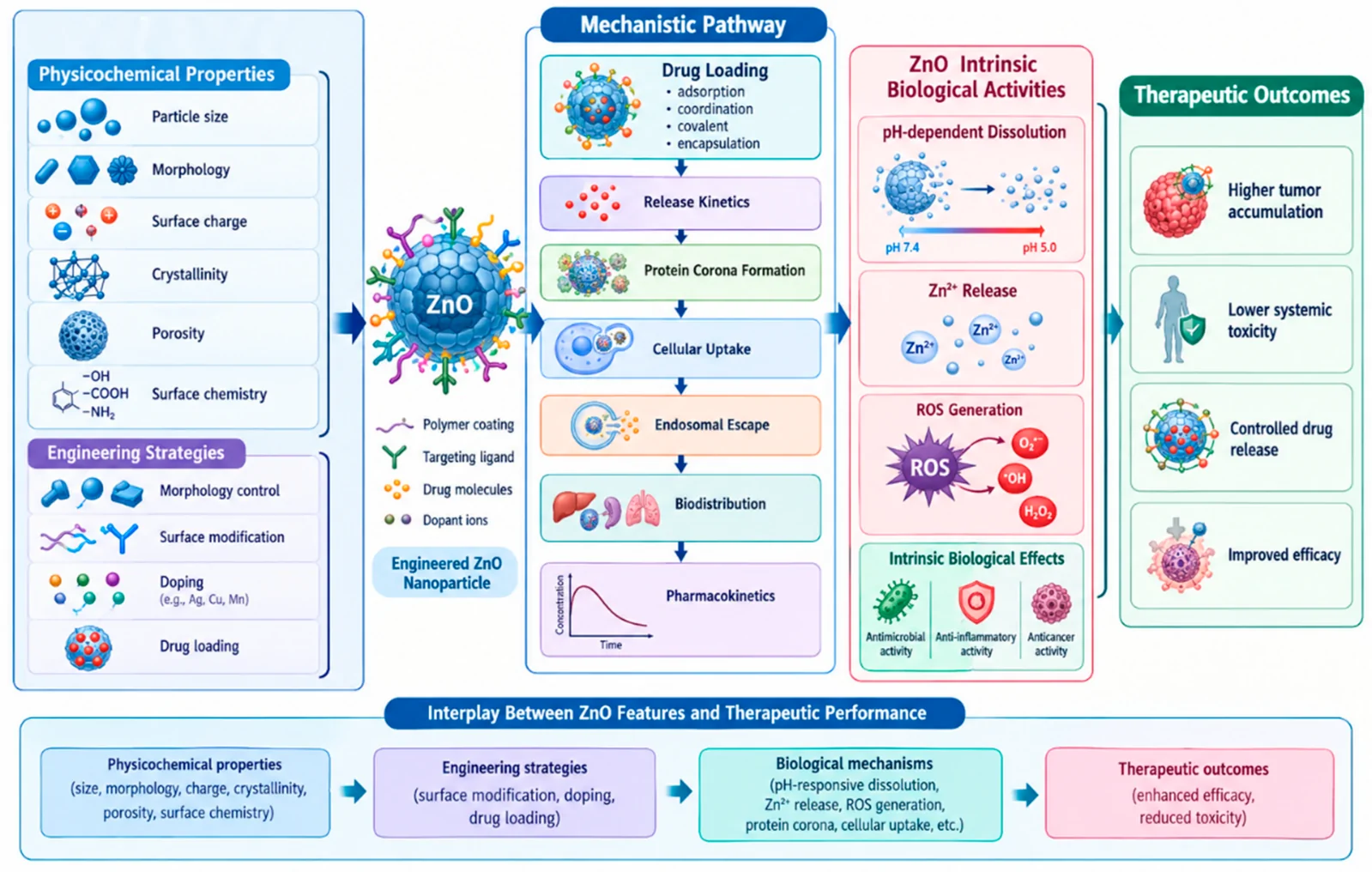
Factors Governing the Therapeutic Performance of ZnO Nanoparticles. Schematic representation of the interplay between ZnO physicochemical properties, nanoparticle engineering, biological mechanisms, and therapeutic outcomes. Particle size, morphology, surface charge, crystallinity, porosity, and surface chemistry influence morphology control, surface modification, doping, and drug-loading strategies, which collectively regulate drug release, protein corona formation, cellular uptake, endosomal escape, biodistribution, and pharmacokinetics. ZnO-specific mechanisms, including pH-dependent dissolution, Zn^2+^ release, and reactive oxygen species (ROS) generation, contribute to intrinsic antimicrobial, anti-inflammatory, and anticancer activities and may complement the pharmacological effects of the delivered drug. The integration of these properties and mechanisms can enhance targeting, controlled drug release, tumor accumulation, and therapeutic efficacy while potentially reducing systemic toxicity.

**Table 1:** Four loading strategies for ZnO nanoparticles.

Strategy	Mechanism	Stability	Release Profile	Advantages	Limitations
Physical Adsorption	Van der Waals, electrostatic, H-bonding	Low	Rapid, non-specific	Simple, quick, no drug modification	Easily displaced, lacks specificity
Coordination/Complexation	Coordination bonding between drug and Zn^2+^	Medium	pH-sensitive, inherently triggered	Specific, pH-responsive without additional engineering	Limited to certain drugs, moderate stability
Covalent Conjugation	Chemical bonding via linkers (amide, ester, disulfide)	High	Controlled, sustained	Predictable, stable, suitable for sustained release	Requires conjugation chemistry, risk of drug inactivation
Encapsulation	Physical embedding in polymer/composite matrix	Very High	Complex (diffusion + matrix degradation)	Highest loading capacity, multiple independent mechanisms	Formulation complexity, additional materials required

## Data Availability

No new data were created or analyzed in this study. Data sharing is not applicable to this article.

## Abbreviations

The following abbreviations are used in this manuscript:

ZnO-NPs/ZNPs	Zinc Oxide Nanoparticles
PEG	Polyethylene Glycol
ROS	Reactive Oxygen Species
EAC	Ehrlich Ascites Carcinoma
IL-6	Interleukin-6
DOX	Doxorubicin
PEI	Polyethyleneimine
PVP	Polyvinylpyrrolidone
Cs/CH	Chitosan
PVA	Polyvinyl Alcohol
PLGA	Poly(lactic-co-glycolic acid)
PLA	Polylactic Acid
D-PAA	Dextran-graft-polyacrylamide
BM-MSC	Bone Marrow-Derived Mesenchymal Stem Cell
CQDs	Carbon Quantum Dots
RGD	Arg-Gly-Asp
HER2	Human Epidermal Growth Factor Receptor 2
BSA	Bovine Serum Albumin
SEM	Scanning Electron Microscopy
ICP-MS	Inductively Coupled Plasma Mass Spectrometry
PBA	Phenylboronic Acid-3-Carboxybenzeneboronic Acid
EDC·HCl	1-(3-Dimethylaminopropyl)-3-ethylcarbodiimide Hydrochloride
NHS	N-Hydroxysuccinimide
